# A novel EB-1/AIDA-1 isoform, AIDA-1c, interacts with the Cajal body protein coilin

**DOI:** 10.1186/1471-2121-6-23

**Published:** 2005-04-29

**Authors:** Hongzhi Xu, Michael D Hebert

**Affiliations:** 1 Department of Biochemistry, The University of Mississippi Medical Center Jackson, MS 39216-4505, USA

## Abstract

**Background:**

Cajal bodies (CBs) are nuclear suborganelles that play a role in the biogenesis of small nuclear ribonucleoproteins (snRNPs), which are crucial for pre-mRNA splicing. Upon nuclear reentry, Sm-class snRNPs localize first to the CB, where the snRNA moiety of the snRNP is modified. It is not clear how snRNPs target to the CB and are released from this structure after their modification. Coilin, the CB marker protein, may participate in snRNP biogenesis given that it can interact with snRNPs and SMN. SMN is crucial for snRNP assembly and is the protein mutated in the neurodegenerative disease Spinal Muscular Atrophy. Coilin knockout mice display significant viability problems and altered CB formation. Thus characterization of the CB and its associated proteins will give insight into snRNP biogenesis and clarify the dynamic organization of the nucleus.

**Results:**

In this report, we identify a novel protein isoform of EB-1/AIDA-1, termed AIDA-1c, that interacts with the CB marker protein, coilin. Northern and nested PCR experiments reveal that the AIDA-1c isoform is expressed in brain and several cancer cell lines. Competition binding experiments demonstrate that AIDA-1c competes with SmB' for coilin binding sites, but does not bind SMN. When ectopically expressed, AIDA-1c is predominantly nuclear with no obvious accumulations in CBs. Interestingly, another EB-1/AIDA-1 nuclear isoform, AIDA-1a, does not bind coilin in vivo as efficiently as AIDA-1c. Knockdown of EB-1/AIDA-1 isoforms by siRNA altered Cajal body organization and reduced cell viability.

**Conclusion:**

These data suggest that specific EB-1/AIDA-1 isoforms, such as AIDA-1c, may participate in the regulation of nucleoplasmic coilin protein interactions in neuronal and transformed cells.

## Background

Studies conducted to understand nuclear organization and function have revealed that the nucleus contains a myriad of dynamic, highly organized domains, territories and bodies [1-3]. The Cajal body (CB) is one such structure and contains the highest concentration of small nuclear ribonucleoproteins (snRNPs) in the nucleus [4,5]. SnRNPs are a vital part of the pre-mRNA processing machinery, necessary for both splice site recognition and the splicing reaction. More precisely, the catalyst for the splicing reaction is probably the small nuclear ribonucleic acid (snRNA) component of the snRNP [6]. The human U1, U2, U4, U5 and U6 snRNAs share the intriguing characteristic in that they contain numerous post-transcriptionally modified nucleotides, namely pseudouridines and 2'-*O*-methyl groups [7]. These modifications appear to be important for efficient splicing of RNA and, in the case of U2, are essential for spliceosomal assembly [8]. In addition to base modification, U1, U2, U4 and U5 snRNAs undergo an elaborate maturation pathway in which they are exported to the cytoplasm. In the cytoplasm, these snRNAs are substrates for the survival of motor neurons (SMN) protein [9]. Mutations in SMN cause the neurodegenerative disorder Spinal Muscular Atrophy [10]. SMN is essential for the assembly of core Sm proteins (B/B', D3, D1, D2, E, F, and G) onto the snRNA [11], and participates in additional modification steps and the import of nascent snRNPs into the nucleus [12,13].

After their transport into the nucleus, newly formed snRNPs accumulate first in the CB and then localize to interchromatin granule clusters, which are also known as speckles [14]. Recent work from the groups of Kiss and Bertrand has shown that CBs contain small guide RNAs, termed small Cajal body-specific RNAs (scaRNAs), which localize exclusively to the CB and guide modifications of U1, U2, U4 and U5 snRNA by pseudouridylation and 2'-*O*-methylation [15,16]. Additional work from this same group demonstrates that these snRNAs are indeed subject to base modification in the CB after import from the cytoplasm [17]. For example, short fragments of Sm snRNAs are modified when they are targeted to the CB, but are not modified when targeted to the nucleolus. Other work has shown that telomerase RNA shares structural similarities to scaRNAs and is also enriched within the Cajal body [18,19]. It is possible, therefore, that CBs may play a role in the assembly of the telomerase holoenzyme in addition to its clear role in snRNA modification. Still additional work implicates CBs in U4/U6 assembly [20,21], U4/U6.U5 tri-snRNP recycling [22], U2 snRNP biogenesis [23] and U3 small nucleolar RNA transport [24].

The marker protein for the CB is considered to be coilin, but the majority (70%) of coilin is actually diffusely localized throughout the nucleoplasm [25]. Although coilin is conserved among vertebrates and homologues are present in plants, a corresponding gene has not been identified in worms and flies [4]. Nevertheless, CB-like structures are present in a variety of insects, including Drosophila [26,27]. The accumulation of snRNPs within CBs is contingent upon coilin [28-30] and characterization of coilin knockout mice demonstrates that inbred strains have significant viability and fertility defects [29]. Cell lines derived from coilin knockout mice do not have CBs, but instead have residual CBs which fail to accumulate snRNPs or SMN [29]. Thus while not an essential protein, coilin likely plays an important role in coordinating the activities of various types of RNA processing machineries into a common nuclear structure [4,31,32].

Coilin has been shown to interact directly with SMN and various Sm proteins and can compete with SmB' for binding sites on SMN [30]. The interaction between coilin and SMN has been found to be contingent upon several arginine/glycine (RG) dipeptide repeats present within coilin [30]. These arginines are symmetrically dimethylated, increasing the affinity of SMN for coilin [33,34]. In the nucleus, SMN localizes in most cell types and cell lines to CBs [1]. Curiously, SMN exists in some cell lines and fetal tissues in discrete nuclear structures termed "gems" (for Gemini of Cajal bodies) [35,36]. The presence of SMN gems correlates with a decrease in coilin methylation [33,34].

It is not yet understood how nascent snRNPs are targeted to the CB for modification. Also unclear is how snRNPs are released from the CB after modification of the snRNA moiety takes place. Moreover, the function of coilin in the nucleoplasm, where the majority of the protein is found, remains elusive. In this work, we identify a novel protein isoform of the *EB-1/AIDA-1 *gene, AIDA-1c, as a coilin interacting protein. Expression analysis demonstrates that AIDA-1c is present in neuronal tissue and cancer cell lines. AIDA-1c competes with SmB' for coilin binding sites and localizes predominantly to the nucleoplasm when ectopically expressed. Other EB-1/AIDA-1 isoforms, in particular AIDA-1a, have been shown to interact with the amyloid-β protein precursor (AβPP) intracellular domain (AID) [37,38]. All known EB-1/AIDA-1 isoforms contain a conserved phosphotyrosine binding (PTB) domain, indicating a role in signal transduction and/or protein:protein interaction [39,38]. Interestingly, AIDA-1a does not interact with coilin in vivo as efficiently as AIDA-1c. These findings suggest that specific EB-1/AIDA-1 isoforms, such as AIDA-1c, may participate in the regulation of nucleoplasmic coilin protein interactions, and thereby indirectly affect CB activity in neuronal and transformed cells.

## Results and discussion

### Isolation of AIDA-1c

To broaden our understanding of Cajal bodies and coilin, we conducted a yeast two-hybrid screen with coilin as bait. We chose a human brain cDNA library for this assay because we are interested in assessing CB protein dynamics in neuronal tissue, which is the cell type primarily affected in Spinal Muscular Atrophy. The C-terminal 214 aa of coilin, which contains the RG box, was the bait for the screen. This region of coilin mediates the interaction between coilin and SMN. Additionally, C214 of coilin is sufficient to interact with Sm proteins [30]. Consequently, other proteins that bind C214 may regulate the interplay between coilin/SMN and coilin/Sm and thus play a role in CB dynamics. The isolation of proteins that interact with this important region of coilin is therefore a key first step towards understanding how RNPs are targeted to, modified within and released from the CB.

Several preys were recovered from the screen, one of which appeared to be a partial coding sequence of an EB-1 protein isoform. Transcription of *EB-1 *is activated in t(1:19) pre-B cell acute lymphocytic leukemia [39]. This translocation produces the chimeric oncoprotein E2a-Pbx1, leading to the aberrant expression of three tissue-specific genes, *EB-1 *among them. Recent work has identified other isoforms emanating from this gene and have found that at least one of these proteins associates with the Amyloid-β Protein Precursor (AβPP) Intracellular Domain (AID) and hence are termed AIDA proteins [38]. Alterations in AβPP processing are thought to underlie the pathology of Alzheimer's disease. All known isoforms of the *EB-1/AIDA-1 *gene contain a phosphotyrosine binding (PTB) domain, including the apparently truncated protein we isolated from the two-hybrid screen. PTB domains were initially characterized as protein interaction modules that bind to regions containing phosphotyrosine [40]. However, these domains have also been found to be important for protein:protein interaction in the absence of phosphotyrosine, possibly serving as a platform for a broad range of associations [41]. In addition to the PTB domain, some isoforms of EB-1/AIDA-1 contain other protein interaction domains such Sterile Alpha Motifs (SAM) [42] and ankyrin repeats [43].

**Figure 1:**
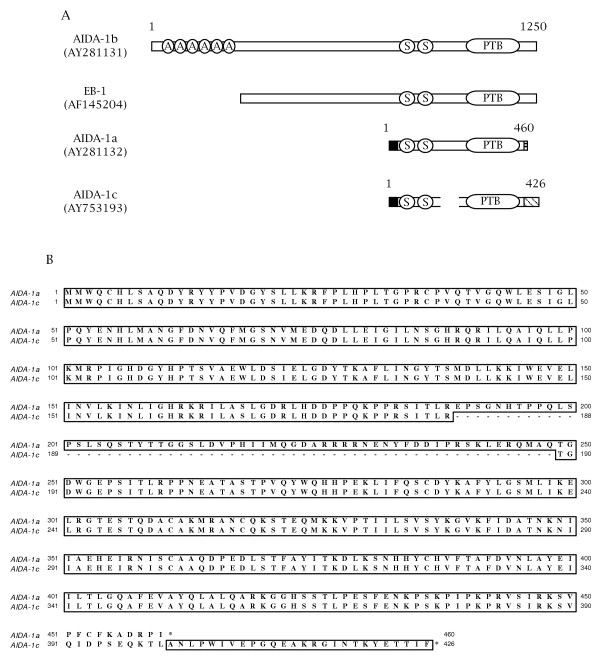
**AIDA-1c is a member of the EB-1/AIDA-1 protein family**. (A) Schematic amino acid alignment of some known EB-1/AIDA-1 isoforms. Accession numbers AY281131 (AIDA-1b) and AY281132 (AIDA-1a) were reported by Ghersi et al., 2004a. AF145204 (EB-1) was reported by [39] and does not contain a translational start site. AIDA-1c (AY753193) is from this study. AIDA-1b is predicted to be approximately 1250 aa and contains ankrin repeats (denoted by 'A'), Eph-related SAM domains ('S') and a phosphotyrosine-binding domain ('PTB'). Boxes with shading or hatching represent variation from the AIDA-1b sequence. (B) Amino acid alignment of AIDA-1a versus AIDA-1c.

Since the cDNA clone obtained from the two-hybrid screen against coilin(C214) appears to be incomplete (it lacks a translational start sequence), we conducted PCR on a human brain cDNA library using a forward primer specific to the translation start of another EB-1/AIDA-1 isoform, AIDA-1a, and a reverse primer specific to the 3' end of the isolated cDNA. Sequencing of the resultant product demonstrated that we amplified a novel EB-1/AIDA-1 isoform, which we have termed AIDA-1c (Figure 1A). AIDA-1c is 426 amino acids length and has a predicted molecular weight of 48 kDa. We have not tested if AIDA-1c binds the AID domain of the amyloid-β protein precursor. Alignment of the protein sequence of AIDA-1c with AIDA-1a demonstrates that the C-terminus of AIDA-1c differs from AIDA-1a (Figure 1B). Furthermore, while the N-terminus of AIDA-1c is identical to AIDA-1a, AIDA-1c contains an internal deletion between the SAM and PTB domains not found in AIDA-1a or other known isoforms. We could not amplify a product that contained the translational start of AIDA-1b and the 3' exon of AIDA-1c.

**Figure 2:**
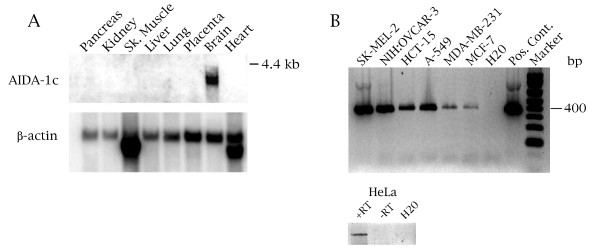
**AIDA-1c expression is highest in neuronal tissue and is present in cancer lines**. (A) A Multi-Tissue Northern blot (Clontech) was probed with an AIDA-1c specific probe. Re-probing of the same blot with a β-actin probe (bottom panel) is shown, verifying the presence of RNA in each lane. (B) (Upper panel) Nested PCR using AIDA-1c specific primers was conducted on cDNA obtained from a variety of cancer cell lines: SK-MEL-2 (melanoma), NIH:OVCAR-3 (ovarian), HCT-15 (colon), A-549 (non small cell lung carcinoma), MDA-MB-231 (breast), MCF-7 (breast). The positive control lane used AIDA-1c cDNA as the template. (Lower panel) HeLa expresses AIDA-1c. HeLa RNA was subjected to RT-PCR using an AIDA-1c specific primer. No product is observed in the reaction lacking reverse transcriptase (-RT).

To get an approximate idea as to the expression profile of AIDA-1c mRNA, we conducted a multi-tissue Northern blot using a probe derived from the unique 3' end of AIDA-1c (Figure 2A). This probe should therefore discriminate between AIDA-1c and other isoforms, unless they share the same 3' exon and untranslated region as found for AIDA-1c. A band of approximately 4 kbp in length corresponding to the AIDA-1c message is detected only in neuronal tissue (recall that a brain cDNA library was used in the isolation of AIDA-1c). To explore if AIDA-1c expression can be detected in cell lines, we conducted nested PCR using primers specific to the 3' end of AIDA-1c on cDNA made from various cancer lines. As shown in Figure 2B, AIDA-1c expression can be detected in all six cell lines. We also observed expression of AIDA-1c in HeLa cells, as assessed by RT-PCR (Figure 2B, lower panel). These results show that AIDA-1c is detected in cancer cells and is expressed at the highest level in brain among normal tissues. However, since nested- and RT-PCR are much more sensitive techniques than Northern blotting, we cannot exclude the possibility that low levels of this isoform are present in normal tissues.

### A 47kDa. protein reactive to AIDA-1 antibodies interacts with the C-terminus of coilin

**Figure 3:**
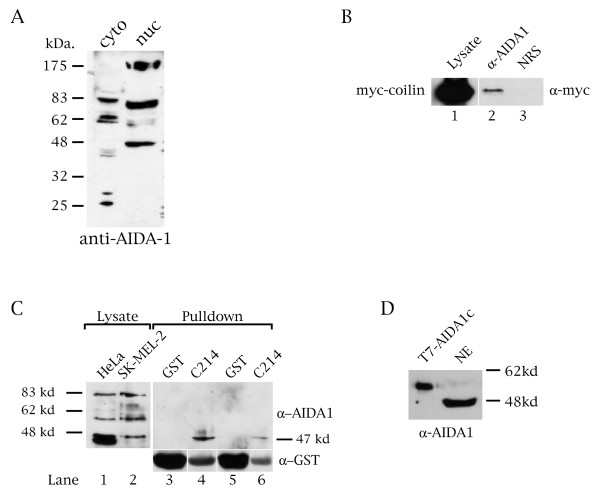
**A 47 kDa. EB-1/AIDA-1 protein interacts with coilin**. (A) Cytoplasmic and nuclear extracts from HeLa cells were subjected to Western blotting with anti-AIDA-1 antibodies. (B) Myc-coilin interacts in vivo with EB-1/AIDA-1 nuclear proteins. HeLa cells were transfected with myc-coilin and nuclear extract was subjected to immunoprecipitation (IP) with anti-AIDA-1 antibodies followed by SDS-PAGE and Western blotting with anti-myc antibodies. Normal rabbit serum (NRS) was used as a negative IP control. The input lane accounts for 1/20 the amount of lysate used in the IP reactions. (C) HeLa and melanoma cell lysates were incubated with a GST-tagged coilin fragment (C214) or GST alone, followed by Western blotting with anti-AIDA-1 antibodies (upper panels). Pulldowns using HeLa lysate are shown in lanes 3 and 4. Melanoma cell lysate was used in the reactions shown in lanes 5 and 6. Only the 47 kDa. EB-1/AIDA-1 protein binds to the coilin fragment. The blot was subsequently probed with anti-GST antibody to verify the loading of the GST and GST-C214 beads (lower panel). The input lanes account for 1/20 the amount of lysate used in the pulldown reactions. (D) Purified T7-tagged AIDA-1c protein was run as a control to mark the position of the 47 kDa. protein from nuclear extract (NE). The blot was probed with anti-AIDA-1 antibody.

Since the two-hybrid result indicates that coilin interacts with a member of the EB-1/AIDA-1 protein family, possibly AIDA-1c, we decided to screen HeLa lysate for EB-1/AIDA-1 proteins that interact with coilin. As a first step, we monitored the expression of EB-1/AIDA-1 proteins from HeLa cytoplasmic and nuclear extract using anti-AIDA-1 antibodies (Zymed, South San Francisco, CA) [37,38]. This antibody was made using a peptide whose sequence is also present in AIDA-1c, and has been confirmed to specifically recognize a number of proteins in human brain extract [37]. By immunoblotting of HeLa lystate, anti-AIDA-1 detects several proteins in both the cytoplasmic and nuclear fractions (Figure 3A). In particular, three prominent proteins, approximately 172, 75, and 47 kDa. in size, are detected by anti-AIDA-1 in nuclear extract. A doublet migrating around 62 kDa. in size is also detected, albeit faintly. To test if any of these proteins interact with coilin, which is also nuclear, we subjected HeLa nuclear lysate to immunoprecipitation with anti-AIDA-1 antibodies, followed by SDS-PAGE and Western blotting with anti-coilin antibodies. We could not detect endogenous coilin association with any AIDA-1 protein (our unpublished results). However, ectopically expressed myc-tagged coilin can be specifically co-immunoprecipitated by anti-AIDA-1 antibodies, but not by normal rabbit serum (Figure 3B). The interaction between coilin and EB-1/AIDA-1 proteins is specific, but likely to be weak or transient given that the input lane accounts for 1/20^th ^the amount of lysate used in the immunoprecipitation reactions (Figure 3B, lane 1).

To corroborate this result and ascertain which EB-1/AIDA-1 protein associates with coilin, we incubated total HeLa or SK-MEL-2 lysate with GST alone or GST fused to the C-terminal 214 aa of coilin. This region of coilin was used in the two-hybrid screen and interacts with Sm proteins and SMN. After extensive washes, the beads were subjected to SDS-PAGE and Western blotting with anti-AIDA-1 antibodies. No proteins reactive to anti-AIDA-1 antibodies were recovered over GST beads, but a 47 kDa. protein was retained by the C-terminal 214 aa coilin fragment in both HeLa and SK-MEL-2 reactions (Figure 3C, lanes 4 and 6 upper panel). Thus the C-terminus of coilin mediates interaction with the 47 kDa. EB-1/AIDA-1 nuclear protein. Of all the known EB-1/AIDA-1 isoforms, only AIDA-1c and a putative protein found in the GenBank database (accession number AF164792) closely match the size of the protein that interacts with the C-terminus of coilin. To confirm that AIDA-1c migrates through SDS-PAGE gels with an apparent size of 47 kDa., we purified a His-T7-tag fusion of AIDA-1c and ran this on a gel with HeLa nuclear extract. Probing with anti-AIDA-1 demonstrates that His-T7-AIDA-1c has a mobility of around 52 kDa. (Figure 3D), indicating that an untagged protein would migrate at 47–48 kDa. (the His-T7 tag and linker add approximately 4 kDa.).

### AIDA-1c interaction with coilin in vivo and in vitro

**Figure 4:**
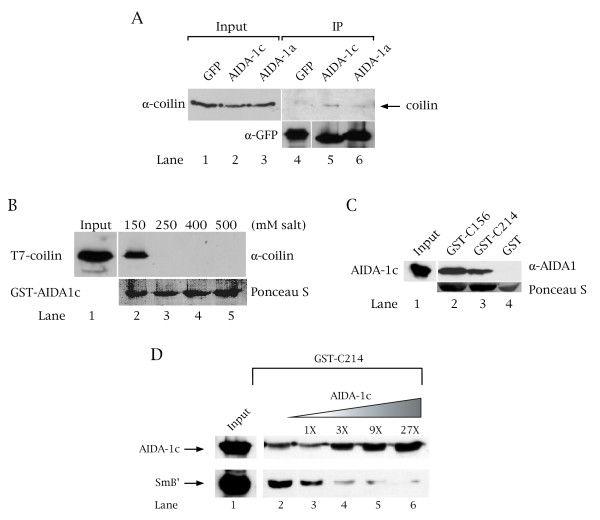
**AIDA-1c associates with coilin in vivo and interacts directly with the C-terminus of coilin in vitro**. (A) Lysates obtained from cells transfected with GFP-only, GFP-AIDA-1c or YFP-AIDA-1a [38] were immunoprecipitated with anti-GFP antibodies followed by Western analysis with anti-coilin antibodies (upper panel). Co-immunoprecipitated coilin can be observed most clearly in the AIDA-1c IP lane (lane 5). The blot was reprobed with anti-GFP antibodies to monitor the expression and immunoprecipitation of the GFP-tagged proteins (lower panel). The anti-GFP signal in lane 4 (bottom panel) has been underexposed six-fold relative to the signal in lanes 5 and 6. The expression level of GFP-AIDA-1c and YFP-AIDA-1a is low, resulting in no signal for these proteins in the input lanes (not shown). Input lanes account for 1/30 the amount of lysate used in the IP reactions. (B) AIDA-1c direct interaction with coilin is salt-sensitive. Purified coilin was incubated with GST-AIDA-1c in binding buffer containing various concentrations of NaCl. After the beads were washed, Western analysis was conducted using anti-coilin antibodies (upper panel). The blot was then stained with Ponceau S to verify that approximately equal amounts of GST-AIDA-1c were used in each reaction (lower panel). The input lane accounts for 20% of the protein used in the pulldown reactions. (C) AIDA-1c directly interacts with the C-terminus of coilin. Purified T7-tagged AIDA-1c was incubated with GST or GST-tagged coilin fragments containing either the C-terminal 156 or 214 amino acids of coilin. The blot was subsequently stained with Ponceau S to verify that approximately equal amounts of GST-fusions were used in the assay (lower panel). The input lane accounts for 20% of the protein used in the pulldown reactions. (D) AIDA-1c competes with Sm proteins for coilin binding sites. A competition experiment is shown wherein individual reactions were set-up containing a fixed amount of GST-coilin fragment (GST-C214) and T7-SmB' with increasing amounts T7-AIDA-1c as indicated. As can been seen in lane 6, as more AIDA-1c binds to GST-C214, less SmB' is recovered. Lane 2 shows the binding of either AIDA-1c (top) or SmB' (bottom) to GST-C214 in the absence of competitor. Input lanes account for 20% of the protein used in the 1X reactions.

To establish if AIDA-1c interacts with coilin, as suggested from the immunoprecipitation and two-hybrid experiments described above, we conducted co-immunoprecipitation experiments on lysate from HeLa cells transfected with empty GFP vector or GFP-AIDA-1c using anti-GFP antibodies followed by Western blotting with anti-coilin antibodies. We also tested another nuclear EB-1/AIDA-1 isoform, AIDA-1a, for its ability to associate with coilin in vivo. As shown in Figure 4A, coilin specifically interacts with GFP-AIDA-1c, but not GFP alone, despite the large amount of immunoprecipitated GFP (the anti-GFP signal in lane 4 (lower panel) has been underexposed six-fold relative to the signals in lane 5 and 6). YFP-AIDA-1a associates with coilin above background levels, but below that found for AIDA-1c (Figure 4A, upper panel, compare the signal in lane 6 to that in lane 5). Reprobing of the same blot verified that both FP-AIDA-1c and FP-AIDA-1a were expressed and immunoprecipitated equally (lower panel, compare the anti-GFP signal in lane 5 to that in lane 6). Therefore, we conclude that AIDA-1a does not form a complex with coilin as efficiently as AIDA-1c.

To determine if the interaction between AIDA-1c and coilin is direct, we conducted GST-pulldown assays using purified recombinant proteins. We found that soluble full-length coilin is recovered by AIDA-1c fused to GST (Figure 4B, lane 2). In support of our previous observations suggesting that the interaction between AIDA-1c and coilin is weak or transient, coilin binding is abolished when the assay was carried out in the presence of increasing salt concentrations (Figure 4B, lanes 3–5). These results indicate that the association between coilin and AIDA-1c is specific and electrostatic bonds are important for this interaction.

To verify that the coilin fragment used in the two-hybrid assay directly interacts with AIDA-1c, we conducted GST-pulldown experiments with bacterially expressed AIDA-1c incubated with GST alone or GST fused to aa 362–576 of coilin (C214). We found that AIDA-1c, detected using anti-AIDA-1 antibodies, interacts directly with the coilin fragment but not with GST alone (Figure 4C, upper panel, compare lane 4 to lane 3). The binding site for AIDA-1c on coilin was further explored using an additional coilin C-terminal fragment (aa 420–576, C156) that lacks the coilin RG box (found between aa 392–420). Recovery of AIDA-1c is observed in the reaction using GST-C156 of coilin (Figure 4C, upper panel, lane 2).

### AIDA-1c competes with Sm proteins for coilin binding

We have previously shown that the C-terminal 214 aa of coilin, in addition to AIDA-1c binding, also mediates the interaction with SMN and Sm proteins, such as SmB' [30]. While the interaction between coilin and SMN involves the coilin RG box, which is subject to modification by symmetrical dimethylation [33,34], the binding site for Sm proteins on coilin has not been defined. However, competition binding experiments show that Sm proteins and SMN may have overlapping binding sites on coilin [30]. Since AIDA-1c binds within the same C-terminal 214 residues of coilin as do SMN and Sm proteins, we investigated whether AIDA-1c may compete with Sm proteins for coilin binding sites. AIDA-1c can effectively compete with SmB' for coilin binding sites (Figure 4D, compare the level of SmB' recovered in lane 2 to that in lane 6). We did not observe a direct interaction between SMN and AIDA-1c (our unpublished observations). Thus, the findings that AIDA-1c competes with Sm proteins and binds downstream of the coilin RG box suggest that Sm proteins and SMN have overlapping but distinct binding sites on coilin. The interaction studies also demonstrate that one function of AIDA-1c may be to modify coilin protein dynamics.

### Cellular localization of ectopically expressed AIDA-1c

**Figure 5:**
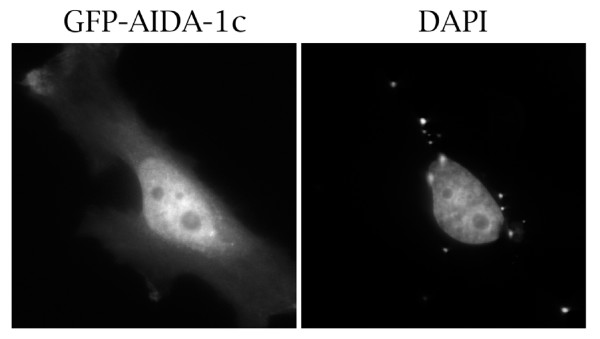
**Ectopically expressed AIDA-1c is predominantly nuclear**. HeLa cells transfected with GFP-fused to AIDA-1c were processed and stained with DAPI. GFP-AIDA-1c is predominantly localized in the nucleus.

Given that AIDA-1c interacts with coilin, and that a 47kDa. protein which is possibly AIDA-1c is found in nuclear extract, we were interested in assessing the cellular localization of AIDA-1c by transient transfection into HeLa cells. As shown in Figure 5, GFP-tagged AIDA-1c is localized diffusely in the nucleus, excluding nucleoli, with some staining in the cytoplasm. We did not detect significant enrichment of AIDA-1c in CBs (our unpublished observations). A similar localization pattern has been observed for AIDA-1a [37,38]. Therefore, interactions between AIDA-1c and coilin may be taking place in the nucleoplasm. Indeed, while coilin is highly enriched in the CB, the majority (70%) of the protein is diffusely localized throughout the nucleoplasm [25]. To determine the localization of endogenous EB-1/AIDA-1 proteins, we stained HeLa cells with antibodies to EB-1/AIDA-1 proteins. Despite the fact that anti-AIDA-1 works well by immunoblotting, this antibody does not detect nuclear proteins in HeLa cells by immunofluorescence [37,38] (our unpublished observations). We are currently developing an antibody to the unique C-terminus of AIDA-1c in order to specifically detect this isoform.

### Knockdown of EB-1/AIDA-1 proteins by siRNA reduces cell viability and disrupts Cajal bodies

**Figure 6:**
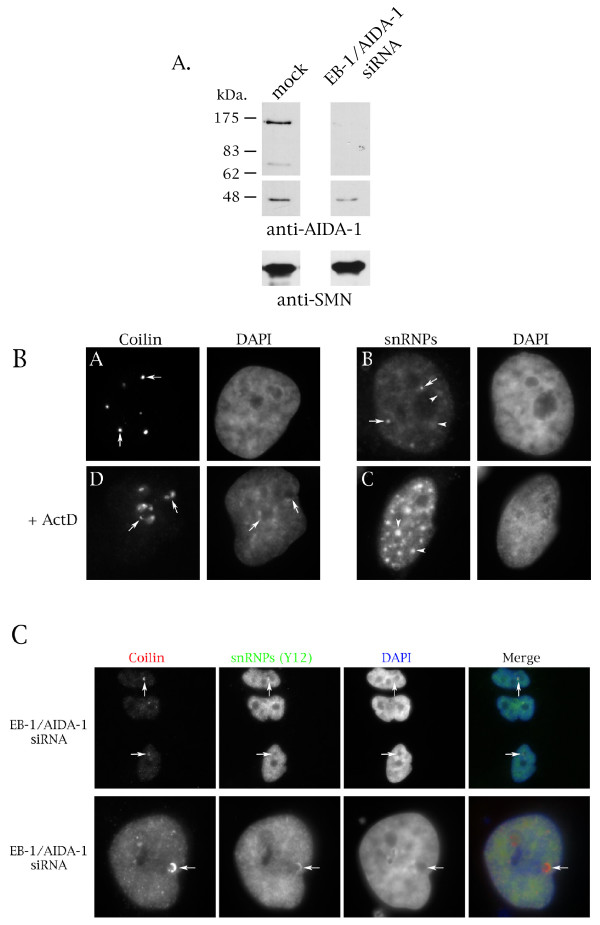
**EB-1/AIDA-1 siRNA disrupts Cajal body structure and causes cell death**. (A) Endogenous EB-1/AIDA-1 protein levels are reduced upon treatment with EB-1/AIDA-1 siRNA. HeLa cells were treated with EB-1/AIDA-1 siRNA (100 nM) for 72 hours, followed by nuclear extraction and Western blotting using anti-AIDA-1 antibodies. The film with the 47 kDa. protein was exposed for a shorter period of time. The blot was reprobed with anti-SMN to verify that equivalent levels of extract were loaded on the gel. (B) Coilin and snRNP localization is disrupted upon treatment with actinomycin D. Panel A shows the normal localization of coilin in CBs (arrows), along with the DAPI stain of the same cell. Panel B shows the normal localization of snRNPs to CBs (arrows) and speckles (arrowheads). Panels C and D are representative images of cells treated with actinomycin D. Note that coilin is relocalized to the periphery of the nucleolus (panel C, arrows) and speckles are enlarged (panel D, arrowheads) in the presence of actinomycin D. (C) HeLa cells exposed to EB-1/AIDA-1 siRNA (100 nM for 72 hours) were subjected to immunofluorescence analysis with antibodies to coilin (red) and snRNPs (Y12) (green). The overlay is shown in the far right panel. Arrows mark altered Cajal body structure. The bottom panels show a closer view of another cell with disrupted CB organization and co-localization of coilin and snRNPs.

The studies described above suggest that a subset of EB-1/AIDA-1 proteins may play a role in Cajal body protein dynamics. To further explore this possibility, siRNA was used to target all known EB-1/AIDA-1 proteins. For this experiment, cells were treated with EB-1/AIDA-1 siRNA for 72 hours, followed by nuclear lysate preparation and Western blotting using anti-AIDA-1 antibodies. Three prominent bands reactive to anti-AIDA-1 are detected in nuclear lysate from mock treated cells 172, 75, and 47 kDa. in size (Figure 6A, top panel). The amounts of these proteins is significantly reduced in extract obtained from cells treated with EB-1/AIDA-1 siRNA. In fact, the 47 kDa. protein, which we believe is AIDA-1c, is reduced by 3.5 fold as assessed by densitometric analysis. Reprobing of the same blot with anti-SMN demonstrates that an approximately equal amount of nuclear protein was loaded in each lane.

During these experiments, we noticed an increased rate of cell death in cells treated with EB-1/AIDA-1 siRNA compared to mock treated. We quantified this phenomenon by conducting experiments with mock-, EB-1/AIDA-1-, or control siRNA-treatment followed by cell counting using the trypan blue exclusion method. The control siRNA used in this study is functional in that it is processed by the RISC machinery, but is non-targeting (Dharmacon Research). Thus this siRNA is an excellent control for off-target effects. In fact, there is no reduction in cell viability when using the control siRNA. In contrast, only 45% (+/- 4%) of cells are viable upon treatment with EB-1/AIDA-1 siRNA.

Since EB-1/AIDA-1 siRNA causes cell death and a subset of EB-1/AIDA-1 isoforms interact with the Cajal body protein coilin, we investigated if nuclear organization, in particular Cajal body structure, was disrupted upon treating cells with EB-1/AIDA-1 siRNA. The normal localization of coilin in CBs is shown in Figure 6B (panel A, arrows), as is the typical distribution of snRNPs in speckles and CBs (panel B, arrowheads and arrows, respectively). In contrast, CBs and speckles display altered structures in the presence of the transcription inhibitor actinomycin D (Figure 6B, panels C and D, [44]). Specifically, CBs relocalize to nucleolar caps (panel C, arrows) and speckles enlarge (panel D, arrowheads). Cells exposed to EB-1/AIDA-1 siRNA have noticeable changes in nuclear organization compared to mock- or control siRNA-treated cells. Notably, coilin and CBs are reorganized in a manner reminiscent to that observed when treating cells with actinomycin D (Figure 6C). In contrast, mock or control treated cells are indistinguishable from normal cells with regard to CB organization (not shown). One noticeable difference between the phenotype of EB-1/AIDA-1 siRNA versus actinomycin D treated cells is that snRNPs, as assessed by Y12 staining, are co-localized with coilin in EB-1/AIDA-1 siRNA cells (Figure 6C, arrows). Previous reports [44] and our work (Figure 6B, panel D) have shown that snRNPs do not co-localize with coilin in actinomycin D treated cells. It is conceivable, therefore, that the observed cell death upon treating cells with EB-1/AIDA-1 siRNA is the result of altered Cajal body organization rather than simply an arrest of transcription. Given that several EB-1/AIDA-1 proteins are likely to be targeted by the EB-1/AIDA-1 siRNA, we cannot ascertain the specific function of AIDA-1c in these experiments. However, if AIDA-1c does participate in snRNP trafficking through the CB, then we would expect to observe altered snRNP transport when AIDA-1c levels are reduced. The presence of snRNPs in coilin-containing nucleolar cap structures formed by EB-1/AIDA-1 siRNA treatment is therefore consistent with a role of AIDA-1c in snRNP displacement from the CB.

## Conclusion

The isolation and partial characterization of AIDA-1c demonstrates that specific EB-1/AIDA-1 protein isoforms may regulate the dynamics of coilin protein interactions in the nucleoplasm. Little is known about the role of nucleoplasmic coilin, even though this fraction accounts for the majority of the protein [25]. For example, coilin could interact with snRNPs in the nucleoplasm and bring them to the CB. Coilin association with AIDA-1c in the nucleoplasm might therefore affect the protein composition of the CB indirectly. If correct, then we would expect that the specific disruption of AIDA-1c expression would alter some aspect of snRNP biogenesis or CB function. Current experiments are underway to test this hypothesis.

It is noteworthy that AIDA-1c expression can be detected in both neuronal and cancer cells given that Cajal bodies are prominent in these cells [1]. Curiously, certain neurodegenerative disorders such as Spinal Muscular Atrophy (SMA) dramatically affect neuronal cells even though the mutant protein (SMN) is ubiquitously expressed. Why is neuronal tissue so susceptible to disease proteins? One possible explanation is that neuronal cells, which are known to undergo a high degree of alternative splicing [45], cannot tolerate deficiencies in splicing. It may be that a range of neurodegenerative disorders, through a variety of mechanisms, affect the level of neuronal alternative splicing by disrupting Cajal body function. Consequently, in addition to other cellular processes which are disturbed in some neurodegenerative disorders, inadequate levels of snRNPs may exacerbate the deleterious effects of the disease protein in neuronal tissue. Interestingly, CBs are not normally found in every human cell but are present in immortalized or transformed cells [36,46]. The induction of CBs in cancer cells may reflect on the need for increased levels of snRNPs. Given the presence of CBs in neuronal and cancer cells, it would not be surprising if various factors were upregulated in these cell types to meet their RNP demands. Indeed, EB-1 mRNA is normally expressed in brain and testis but is dramatically induced in marrow from patients having a specific acute lymphocytic leukemia [39]. Likewise, we have found that AIDA-1c expression can be detected both in brain and certain cancer cell lines. We propose that AIDA-1c expression in neuronal and cancer cell lines may facilitate snRNP biogenesis by modulating the interaction between coilin and snRNPs.

## Methods

### Yeast two-hybrid screen and plasmid construction

A human brain cDNA library cloned into the prey vector pACT2 and pretransformed into the yeast strain Y187 (Clontech) was mated with the strain PJ69-2A harboring the bait vector pAS2-1-coilin(362–576, C214) per the manufacturer's instructions. After mating, the yeast were plated onto medium lacking tryptophan, leucine, histidine and adenine (to select for bait, prey and protein interaction). Colonies were picked after several days of incubation and the prey plasmid was isolated and transformed into PJ69-2A containing pAS2-1-C214 or control bait plasmids to confirm the specificity of the interaction. Restriction digests and sequencing revealed that nine different prey cDNAs were recovered, one of which appeared to encode a truncated EB-1/AIDA-1 protein and was termed CAP36C. The sequence for CAP36C has been submitted to GenBank and given the accession number AY356353. For bacterial expression, cDNAs were cloned into pET28a (Novagen), generating a His-T7-tag fusion, or pGEX-2T/ pGEX-3X (Amersham Pharmacia), generating a fusion to GST. Additional constructs were generated employing standard molecular biological techniques or have been described previously [47,30,33].

### Isolation of AIDA-1c

AIDA-1c was isolated by conducting PCR on a human brain cDNA library (Clontech) using a forward primer specific to the translation start of another EB-1/AIDA-1 isoform, AIDA-1a (accession number AY281132), and a reverse primer specific to the 3' exon of AIDA-1c. The sequence for AIDA-1c has been submitted to GenBank and given the accession number AY753193. AIDA-1c was cloned into various expression vectors by standard molecular biological techniques. We could not amplify a product that contained the translational start of AIDA-1b and the 3' exon of AIDA-1c.

### Northern analysis

A Multi-Tissue Northern blot (Clontech) was hybridized with a ^32^P-radiolabled cDNA probe for AIDA-1c according to the manufacturer's instructions. The probe was generated by PCR using CAP36C DNA as template and primers designed to amplify a fragment of AIDA-1c downstream of the PTB domain, including the 3' untranslated region.

### Nested- and RT-PCR

The Nested PCR assay was carried out in the DNA Engine PTC-200 Peltier Thermal Cycler (MJ Research, Watertown, MA) to assess the expression of AIDA-1c in cultured cancer cell lines. The cDNA from six cancer cell lines were kindly provided by Dr. Laree Hiser (The University of Mississippi Medical Center): SK-MEL-2 (melanoma), NIH: OVCAR-3 (ovarian cancer), HCT-15 (colon cancer), A-549 (non-small cell lung cancer), MDA-MB-231 (breast cancer) and MCF-7 (breast cancer). Two pairs of nested primers flanking the AIDA-1c specific sequence on the 3' end of the AIDA-1c cDNA were used to amplify the sequence. Plasmid containing the AIDA-1c cDNA and water served as positive and negative controls for the reaction, respectively. Each reaction had 30 cycles and, after the first reaction, the product was diluted 50 fold to serve as the template for the second pair of primers. For RT-PCR reactions, DNA-free RNA was isolated from HeLa cells using the RNAqueous-4PCR DNA-free RNA isolation kit (Ambion, Austin, TX). The RNA was reverse-transcribed with the Thermoscript RT-PCR kit (Invitrogen) using an antisense primer for 45 minutes at 50°C. The antinsense primer binds the 3' UTR of AIDA-1c. A forward primer which binds to the 3' coding sequence of AIDA1c was used in combination with the antisense primer to amplify the corresponding cDNA frangment (25 cycle reaction).

### Antibodies

A polyclonal anti-AIDA-1 antibody was purchased from Zymed (South San Francisco, CA) A monoclonal antibody to coilin [48] was a gift from Greg Matera (Case Western Reserve University). We also utilized a commercially available coilin mnonclonal antibody (ab11822) from Abcam (Cambridge, MA). Anti-T7 (1:1000) was obtained from Novagen (Madison, WI). Antibodies to GFP were from BD Biosciences, Palo Alto, CA (polyclonal, catalog number 632459) or Roche, Indianapolis, IN (monoclonal, catalog number 1814460). Myc and GST antibodies were obtained from Santa Cruz Biotechnology, Santa Cruz, CA

### Cell Culture, Transfection, Immunofluorescence, and Immunoprecipitation

HeLA cells were cultured, transfected with Superfect (Qiagen, Valencia, CA) and processed as described [47,30,33]. For immunofluorescence, cells were grown on chamberslides and fixed with 4% paraformaldeyde followed by permeabilizaton with 0.5% Triton X-100. The permeable cells were then blocked with 10% normal goat serum for 30 min and probed with the corresponding primary and secondary antibodies for 30 min each. For co-immunoprecipitation experiments, the protocol detailed in [47] was followed with a few modifications. Briefly, cells were lysed in a modified RIPA buffer (50 mM Tris, pH 7.5, 150 mM NaCl, 1% NP-40, 1% sodium deoxycholate, 0.1% SDS, 2 mM EDTA plus 1 tablet of complete protease inhibitor cocktail (Roche, Mannheim, Germany) per 50 ml lysis solution). The lysates were then repeatedly passed through a 25 gauge needle to shear DNA or passed through a Qiashredder column (Qiagen) and subject t incubation with the appropriate antibodies for 1 hour at 4°C with gentle shaking. Following the addition of protein A or G Sepharose (Amersham Pharmacia, Uppsala, Sweden) for 1 hour with gentle shaking, the beads were washed with 1 ml lysis solution 5 times. The beads were resuspended in 5X SDS loading buffer, boiled, and subject to SDS-PAGE and Western blotting as described [47]. Input lanes typically account for 1/20 to 1/50 the amount of lysate used in the immunoprecipitation reactions. For co-immunoprecipitation experiments on nuclear protein, nuclear extract was made using the NE-PER kit from Pierce, followed by dilution of the extract to 1 ml with mRIPA and immunoprecipitaton as described above. Where indicated, actinomycin D was used at 2.5 μg/ml for two hours.

### In vitro binding assays

GST- and His-tagged constructs, after transformation into *E. coil *BL21(DE3)pLysS cells, were indiced and purified as described [30]. In a binding reaction, approximately 1 ug of His-T7-tagged protein was incubated with 1 ug of the GST-fusion protein in 1 ml of mRIPA plus 2 mM DTT. After incubation for 1 hour at 4°C with gentle inversion, the beads were washed 5 times (1 ml each) with mRIPA plus DTT, resuspended in 15 ul 5X SDS loading buffer, boiled and subjected to SDS-PAGE. Anti-T7 (1:1000; Novagen, Madison, WI) was used to detect SmB' and anti-AIDA-1 was used to detect AIDA-1c. Competition experiments were performed as described in [30], except that increasing amounts of AIDA-1c were used. Input lanes account for 1/5 the amount of lysate used in the pulldown reactions. Additional NaCl was added to the binding buffer where indicated.

### SiRNA knockdowns and viability assays

SiRNAs to the clone obtained in the two-hybrid screen described above (accession number AY356353) were obtained from Dharmacon Research, as was control siRNA (no. D-001210-01-05). The siRNA should target all known EB-1/AIDA-1 isoforms. SiRNA was introduced into HeLa cells using Lipofectamine 2000 per the manufacturers' directions (Invitrogen). To assess the knockdown of endogenous EB-1/AIDA-1 proteins, EB-1/AIDA-1 siRNA was added to cells at a final concentration of 100 nM, followed by 72 hours of incubation. Nuclear extract was then made using the NE-PER kit from Pierce, followed by Western blotting with anti-AIDA-1 and anti-SMN antibodies. Densitometric analysis of the films was conducted using ImageQuant software. To monitor the viability of cells treated with EB-1/AIDA-1 siRNA, equal numbers of cells were seeded into three wells of a six-well plate. One well was mock treated (no siRNA), while the other two received 100 nM of control or EB-1/AIDA-1 siRNA. After 72 hours of incubation, the cells were counted and scored for viability using Trypan blue. The experiment was done in triplicate.

## Authors' contributions

HX made the DNA constructs and conducted most of the experiments. MDH conceived the experiments, coordinated the study and wrote the paper.
